# ACHESS – The Australian study of child health in same-sex families: background research, design and methodology

**DOI:** 10.1186/1471-2458-12-646

**Published:** 2012-08-13

**Authors:** Simon Robert Crouch, Elizabeth Waters, Ruth McNair, Jennifer Power, Elise Davis

**Affiliations:** 1 The McCaughey Centre, Melbourne School of Population Health, The University of Melbourne, Level 5, 207 Bouverie St, Carlton, VIC, 3053, Australia; 2 The Department of General Practice and Northwest Academic Centre, The University of Melbourne, 200 Berkeley St, Carlton, VIC, 3053, Australia; 3 The Bouverie Centre, La Trobe University, 8 Gardiner St, Brunswick, VIC, 3056, Australia

**Keywords:** Child health, Gay parents, Lesbian parents, Same-sex parents, Homosexuality, Sexual orientation

## Abstract

**Background:**

There are an increasing number of children in Australia growing up with same-sex attracted parents. Although children from same-sex parent families do in general perform well on many psychosocial measures recent research is beginning to consider some small but significant differences when these children are compared with children from other family backgrounds. In particular studies suggest that there is an association between the stigma that same-sex parent families experience and child wellbeing. Research to date lacks a holistic view with the complete physical, mental and social wellbeing of children not yet addressed. In addition, most studies have focused only on families with lesbian parents and have studied only small numbers of children.

**Methods/design:**

The Australian Study of Child Health in Same-Sex Families (ACHESS) is a national study that aims to determine the complete physical, mental and social wellbeing of Australian children under the age 18 years with at least one parent who self identifies as being same-sex attracted. There will be a particular focus on the impact that stigma and discrimination has on these families. Parent and child surveys will be used to collect data and will be available both online and in paper form. Measures have been chosen whenever possible that have sound conceptual underpinnings, robust psychometric properties and Australian normative data, and include the Child Health Questionnaire (CHQ), the Strengths and Difficulties Questionnaire (SDQ) and the Kessler Psychological Distress Scale (K10).

**Discussion:**

ACHESS aims to be the largest study of its kind and will for the first time produce a detailed quantitative analysis of Australian children with same-sex attracted parents. By inviting participants to take part in further research it will also establish a valuable cohort of children, and their families, to launch future waves of research that will help us better understand the health and wellbeing of children with same-sex attracted parents.

## Background

Conservative estimates show that in 2011 there were more than 33,000 same-sex couples living together in Australia, an increase from around 19,000 ten years earlier [1,2]. The number of children living in these families almost doubled over the same period and it is now estimated that that there are around 6,120 children under the age of 25 years living with two same-sex parents [1]. These figures do not capture children living with same-sex attracted single parents, or parents who are reluctant to self-identify as same-sex attracted due to fear of stigma and discrimination. The recent Private Lives 2 report, a national survey of the health and wellbeing of gay, lesbian, bisexual and transgender Australians, identified that 22.1% of respondents reported having children or step children [3]. In the Australian context ongoing reforms allowing same-sex adoption and increased access to surrogacy and fertility treatments for same-sex couples [4] suggest that the number of children growing up in same-sex parent households is also likely to grow.

Same-sex attracted adults are known to be at increased risk of psychological disorders, and it has been suggested that discriminatory policies may be associated with mental health outcomes for this population [5]. Furthermore, experiences of discrimination vary depending on where they live [6,7]. Given that same-sex attracted people are increasingly raising children it is important to determine how this context impacts on child health and wellbeing. This paper aims to highlight the findings from research to date on the health and wellbeing of children with same-sex attracted parents and describes an Australian study that aims to measure the health and wellbeing of children aged 0–17 years, with a particular focus on the dimensions of importance to children within this familial context.

In order to define the conceptual framework and population context, we have selected the World Health Organization definition of health, where health is “the complete physical, mental and social wellbeing of children and not merely the absence of disease or infirmity” [8]. In relation to the potential population denominator, we have defined same-sex families for the purpose of this research to maximize comparability internationally, as ‘any family in which at least one parent is same-sex attracted’.

### Understanding difference

Over the last two decades reviews of the literature from Northern Europe and the United States on the health and wellbeing of children with same-sex attracted parents have suggested that there is no difference when these children are compared to children from other family backgrounds with respect to social, emotional, developmental and educational outcomes; the so called ‘no difference consensus’ [9-11]. Stacey and Biblarz in 2001 were among the first to argue that a closer inspection of the literature identifies a number of areas that do not immediately follow the generally accepted ‘no difference’ hypothesis [12], including child sexual orientation and gender role behavior [13-21]. Health care professionals no longer view homosexuality as a negative health outcome however. While conservative aspects of society may dispute this, as do some authors in the field [16], other authors on child health and wellbeing in same-sex families maintain that child sexual orientation is not a marker of quality of parenting [12]. In fact, it has been argued that asking a question that compares the sexual orientation of children with same-sex parents to children with heterosexual parents reinforces a heteronormative viewpoint that stigmatises same-sex families further [22].

### Stigmatisation

Increasingly research on child health and wellbeing in same-sex families has begun to consider stigma and discrimination. The findings from this research demonstrate that the experiences of stigmatisation is one area that is consistently associated with the health and wellbeing of children with same-sex attracted parents [9,15,17,23-27]. Frequently, studies have found that when there is perceived stigma, experiences of rejection or homophobic bullying, children with same-sex attracted parents are more likely to display problems in their psychosocial development [7,14,28-32]. The experience of discrimination is by no means universal. Bos et al’s 2008 study comparing children in the US and the Netherlands suggests that children in the US sample experience much more homophobia than the Netherlands and that this homophobia is related to higher levels of problem behaviors by comparison to the Dutch sample [7]. This cultural context appears to play an important role. In 1993 Javaid showed that US children with heterosexual parents display high levels of prejudice towards homosexuality, and Gershon et al. in 1999 demonstrated that in the USA there is a strong link between perceived stigma and poor self-esteem for children with same-sex parents [14,30]. Interestingly however, countries that are considered to be more liberal in their attitudes towards homosexuality (the United Kingdom, the Netherlands, Belgium and Canada – all of which currently allow same-sex marriage or civil partnerships) do not appear to identify as many significant differences in teasing between children with same-sex attracted parents and children with heterosexual parents [33-35]. Currently Australia does not recognise same-sex marriages, and Queensland has recently wound back legislation to remove civil partnerships from that state’s statutes [36]. How this lack of political recognition of same-sex families impacts on the stigma perceived by children is not yet known. Of some concern however is research from 2001 conducted by Ray and Gregory. They found high levels of bullying experienced by Australian children from same-sex families. Much more detailed investigation is required however, taking into account different socioeconomic and cultural settings [31].

### A holistic view

When considering the health and wellbeing of children with same-sex attracted parents 40 relevant studies were conducted between January 1990 and March 2011, the first by Javaid in 1993 [14] and the most recent a report from the fifth wave of the National Longitudinal Lesbian Family Study in the USA [37]. Australian research from 2008 has suggested that lesbian parents perceive barriers when dealing with the healthcare system [38]. Given the benefits of effective interactions with health care providers, particularly in the very early years of childhood where prevention and early intervention, continuity of care and integration of services are central [39], it is important to establish whether potential barriers, which might include perceived stigma, have an impact on the physical wellbeing of children with same-sex attracted parents.

Of the 40 studies described above only five have considered physical aspects of health and wellbeing and these have only presented crude figures pertaining to a handful of common childhood ailments [40]. It is clear that a more comprehensive approach to understanding health and wellbeing is required to fully capture the complete health experience of children with same-sex attracted parents.

### Parental gender

The influence of parental gender and parenting has been another area of enquiry, and in this context Stacy and Biblarz, who reviewed the existing literature in 2001, suggest that, rather than sexual orientation, parental gender may play a role in child health and wellbeing, highlighting research that scores both lesbian and heterosexual mothers better in measures of effective parenting than heterosexual fathers [12]. They argue that mothers are more emotionally invested in raising children than fathers are in general, which has been supported by other authors [41-43] and includes research by Gatrell (2005, 2010) and Golombok (2010) [40,44,45]. The research conducted by Golombok et al. in 1997 suggests that absent fathers may be detrimental to self-rated cognitive and physical competence [46]. One area that many authors are in agreement on however is that there is a lack of research looking at male same-sex parented families [47]. While it cannot be assumed that the findings described by Stacey and Biblarz suggest children with gay male parents would perform poorly in their health and wellbeing outcomes, Cameron (2009) argues that all too often authors erroneously extrapolate results from research on lesbian parenting to the whole range of same-sex families [48] and as such outcomes from lesbian headed families should not be directly applied to gay male families. Only seven studies have considered gay fathers. One of these studies, conducted in 2010 in the Netherlands, compared children with gay biological fathers to children with heterosexual, married, biological fathers and found that overall there was no difference in child wellbeing. When the gay fathers perceived stigma however their children did show poorer psychological outcomes [49]. It should be noted that this research looked at families where gay men donated sperm as part of a kinship arrangement and the children were not being raised in mother absent families. To date there is no substantial research looking at the health and wellbeing of children residing from birth with gay male parents and conclusions cannot therefore be drawn about their health and wellbeing in this setting.

### Representing the population

Overall, the 40 studies suggest that many of the factors that might be expected to have an influence on child health and wellbeing hold true for children with same-sex attracted parents, such as family relationships - parental relationships with each other and their children; family income; parental education; and socioeconomic status [10,44,50,51]. It is often assumed that in developed countries same-sex parent families are well educated and fall into higher socioeconomic status groupings. This assumption is supported by much of the literature that has recruited families using convenience sampling techniques. The few studies that have been able to employ a more representative sampling method, identifying same-sex parent families from broader population surveys, have found no difference in income, SES and years of education when comparing the same-sex parent families to the heterosexual parent families [34,52-54]. One study even suggests that same-sex parent families may have lower income, SES and fewer years of education [55]. It is these factors, set in a broader social context, that require further study to elicit the effect of the lived environment on the health and wellbeing of children with same-sex attracted parents. In the Australian setting this would involve seeking participation from ethnic minority and rural same-sex attracted parents, and those from low socioeconomic groups.

### Methodological issues

It has been argued that there should be a greater focus on child health and wellbeing in the context of same-sex parent families with a number of recent studies no longer making direct comparisons to heterosexual parent families [15,31,37,56]. While it is important to identify how the children in these families are fairing and what factors ensure their optimal health and wellbeing, measuring health and wellbeing using instruments that have population level normative data gives a baseline from which epidemiological analysis can evolve. Taking these factors into account it is suggested that the focus should be on issues relating to stigma, as this has been shown to have a significant impact on health and wellbeing [29].

Most of the quantitative studies utilise standardised tools for data collection, although there is a wide variety in the specific tools chosen. The only tool to have been used widely, across differing research collaborations, is the Child Behavior Check List (CBCL) [35,37,43,44,50,51,57]. Recent research has suggested that other tools, such as the Strengths and Difficulties Questionnaire (SDQ), can effectively provide substantial added value when compared with the CBCL and may be preferred by both parents and preventive child health care professionals [58]. None of the tools used however have allowed detailed measurement of physical wellbeing and as such other validated instruments should be considered for use in conjunction with those described to date in the literature.

### The current study

In light of the above research, its strengths and limitations, the questions that remain unanswered relate to understanding the multidimensional experience of physical, mental and social wellbeing of children with same-sex attracted parents, as well as the complexity of the family contexts and social and physical environments in which children are based. It is essential that the measurement instruments are standardised, gold standard and the recruitment methods are comprehensive and transparent. We would add that the issues of stigma and discrimination for these children and their families is a new dimension that hasn’t adequately been captured and a contemporary exploration of this and its facets needs to be included.

Every effort should be made to recruit a diverse sample from the broad range of all families in the gay, lesbian, bisexual and transgender community to ensure maximum representation, and the possibility to extrapolate results to the wider context. In particular attempts should be made to recruit gay male parents and their children, as this growing sub-population requires much greater consideration.

## Methods/design

### Aim and objectives

The Australian Study of Child Health in Same-Sex Families (ACHESS) is a national study that aims to determine the complete physical, mental and social wellbeing of Australian children with at least one same-sex attracted parent, and to identify factors that may be associated with child health and wellbeing in this setting. In particular, the impact of stigma and discrimination on overall wellbeing will be assessed. The following objectives have been defined:

· To describe the characteristics of families with at least one same-sex attracted parent.

· To measure the complete physical, mental and social wellbeing of children with at least one same-sex attracted parent.

· To investigate the role of stigma and discrimination in the development of health outcomes in children from same-sex families.

· To contextualize the findings from this research into the broader social milieu and to consider potential policy agendas, both locally and nationally, that will optimize the health and wellbeing of children with same-sex attracted parents.

### Design

ACHESS will be conducted between 2012 and 2014. This initial phase of the study will use a questionnaire for same-sex attracted parents to establish the health and wellbeing of all their children under the age of 18 years. Parents can also invite any of their children aged 10 to 17 years to complete a child-version of the questionnaire.

### Setting and participants

The initial sample will comprise children from 2 months to 17 years of age who have at least one self-identified same-sex attracted parent who resides in Australia. This is a national study and will include residents from all states and territories in Australia. Parents will be recruited in the first instance with one parent providing data for each child. All children in a family, under the age of 18 years, will be included in the sample. There are no adequate data available to estimate the total population size for all children in Australia with same-sex attracted parents. As such it is not possible to conduct sample size calculation for the purpose of this study. Looking at recent work with this population, using similar recruitment techniques, it is reasonable to expect that up to 750 children may take part in our study from around 400 families [59].

### Recruitment

Initial recruitment will involve convenience sampling and snowball recruitment techniques that have been successful in other survey-based Australian studies of same-sex attracted populations including the Work, Love, Play Study and the Lesbian and Gay Families Study [59,60]. This will include advertisements and media releases in gay and lesbian press, flyers at gay and lesbian social and support groups, and investigator attendance at gay and lesbian community events. Discussion pieces and interviews with mainstream media outlets will help target families not engaged with the gay and lesbian community, as well as rural and remote families. Primarily recruitment will be through emails posted on gay and lesbian community email lists aimed at same-sex parenting. This will include, but not be limited to, Gay Dads Australia and the Rainbow Families Council of Victoria. Any parent over the age of 18 years, who self-identifies as being same-sex attracted, lives in Australia, and has children under 18 years of age will be eligible to participate in the study. Children aged ten years or over will also be asked to complete a questionnaire.

### Data collection and the ACHESS survey

The survey will be available for completion online with a paper-based version available upon request. The parent report questionnaire will be completed for all children in a family with appropriate questions based on the number and ages of those children. The survey contains almost 150 items and it will take about 20 minutes to complete for each child. For each child 10 years or over parents will be asked to provide consent and contact details if they are happy for them to participate in the research. All parents will also be asked to provide their own contact details if they are willing to take part in future research. It is made clear in the participant information that both of these items are voluntary and they could remain completely anonymous if they wish.

The child report questionnaire will be completed by children aged 10 years and over when contact details have been provided. There are around 100 items and it will take between 20 and 30 minutes to complete.

### Measures

Both surveys cover a range of topics to assess the overall health and wellbeing of children. Where possible questions were drawn from currently available survey instruments that have sound conceptual underpinnings, robust psychometric properties and Australian normative data. A summary of these instruments and their psychometric properties can be seen in Table 1. Items were grouped in the ACHESS survey in the following way:

**Table 1:** Overview of the published psychometric properties for each of the included scales

**Questionnaire or scale**	**Validity**					**Reliability**		
	**Content**	**Criterion**	**Construct**			**Internal consistency (Cronbach’s alpha)**	**Repeatability**	
			**Convergent**	**Discriminant**	**Factor analysis**		**Test- retest**	**Inter-rater**
							**(ICC)**	**(Correlation)**
CHQ								
*Parent report*	+	+*	+*	+*	+*	0.60-0.93*	0.49-0.78* (at 2 weeks)	
							0.47-0.82* (at 6 weeks)^a^	
								0.42-0.64*
*Adolescent report*	+	+*	+*	+*	+*	0.79-0.90*		
ITQOL	+	+	-	+	-	0.73-0.92	0.42-0.82	-
SDQ								
*Parent report*	+	+*	-	+	+*	0.67-0.80*	0.61-0.77* (at 1 year)	
						0.59-0.82*	0.52-0.85* (at 2 weeks)	
								0.32-0.46*
						0.59-0.75* (11+)	0.66-0.82* (at 2 weeks)	
*Child report*	+	-	-	-	-	0.55-0.73* (<11)		
BSS								
*Adult report*	+	-	-	-	-	0.74	-	-
*Child report*	+	-	-	-	-	0.79	-	-
K10	+	+*	-	-	+*	0.92*	0.8123*	N/A

+ Psychometric property tested/identified.

- Psychometric property not tested/not identified.

N/A Psychometric property not applicable.

* Australian data.

^a^ Excludes physical functioning (0.05) and role-social physical (0.08).

### Child health and wellbeing

Health related quality of life (HRQOL) is considered to be a subdomain of the more global concept of Quality of Life (QOL). Although related to functional status, which asks what individuals can do, HRQOL is more interested in how respondents feel about what they can do [61]. In order to capture all aspects of the complete physical, mental and social wellbeing of children the ACHESS survey will incorporate components that determine HRQOL, health, functional status and health related behaviors that all combine to describe the overall wellbeing of participants. To ensure a robust methodology it is important to use psychometrically tested tools with Australian population norms wherever possible. For the ACHESS survey it is important to utilize an instrument that has both parent and child report forms. By drawing on these conceptual underpinnings and psychometric properties only the Child Health Questionnaire (CHQ) has been found to be suitable for our needs [62-67]. To further support the use of the CHQ an additional, complimentary instrument, the Infant Toddler Quality of Life Questionnaire (ITQOL), is available to measure health and wellbeing in children aged 0–5 years [68].

To supplement the CHQ/ITQOL questions a suitable instrument that focuses on psychosocial aspects of child health was sought. The SDQ is less than one quarter of the length of the CBCL and allows greater expediency in completion [69]. It has also been found to be comparable to, or better than, the CBCL when compared with standarised semi-structured interviews [70]. These factors have led to increasing popularity of the SDQ when compared with other similar instruments [58,71].

### Stigma

Bos et al. have developed a stigmatisation scale specifically for use in the context of same-sex families with both parent and adolescent versions [72-74]. These scales were developed and psychometrically tested for use with Dutch lesbian mother families. The use of the Bos stigmatisation scales in this study will be the first time they have been used in the Australian context and across a diverse range of same-sex families. Although good reliability has been demonstrated for both the scales in their original format it should be acknowledged that transferring them to our study population will affect their psychometric performance. Furthermore minor adaptations have been required to ensure that the wording of the scales is applicable to all same-sex families and not just those with lesbian mothers.

 Items relating to the settings of discrimination were drawn from the Work Love Play study [59] and focus on services identified in the Longitudinal Study of Australia’s Children [75].

### Parental physical and mental health

As an important determinant of child health and wellbeing, it is necessary to include an instrument that is able to give a reliable measure of parental mental health. Most commonly used across Australia is the Kessler Psychological Distress Scale (K10). This tool has undergone psychometric testing in Australia and has good normative data for Australian adults [76].

The CHQ contains items relating to parental physical wellbeing that will give a broad overview of parental health and wellbeing.

### Parental status and family structure

Questions about methods of conception and parental relationships were drawn from the Work Love Play study [59] and are designed to identify the full range of family types in the sample population. These items asked about relationship status, both currently and at the time of conception/fostering/adoption, how long parents have been together and whether they cohabit or not. They also identify donor status for children born through assisted reproductive technologies, the use of surrogates and any co-parenting arrangements that may be in place.

### Sociodemographic variables

As well as measuring child health and wellbeing, the survey will collect data on sociodemographic characteristics that may be associated with child health. These items have been drawn from other relevant surveys of child health and wellbeing including the Victorian Child Health and Wellbeing Survey [77] and the Work Love Play study [59]. Parent gender, parental sexual orientation, place of residence, place of birth, ethnicity, occupation and education are all covered. Child gender, current year level and place of birth are included as additional child variables.

### Analysis

Data analysis will begin by using descriptive statistics to map the variety and structure of same-sex families in the sample. Group level comparisons of mean scores with Australian normative data will be made in the first instance using t-tests. Sub group analysis based on child age, child gender and family type will also be conducted depending on sample sizes. The Child Health Questionnaire requires a sample size of between 119 and 294 (depending on the particular scale) to detect a clinically relevant five-point difference accepting a 5% false rejection rate and 80% statistical power, assuming a non-directional hypothesis. Leading on from this, likely age groupings will be 0–4 year olds, 5–9 year olds and 10 years and over.

Further data analysis will use analysis of variance methods to assess the impact of stigma, as measured by the Bos stigmatisation scales, and other sociodemographic factors, on mean scores of child health and wellbeing from the CHQ, ITQOL and SDQ.

### Ethics

This research project has received ethical approval from the University of Melbourne Health Sciences Human Ethics Subcommittee, ethics ID number 1136875.1. Informed consent will be obtained for all participants, including parental consent from all parents of participating children.

Issues around disclosure play a particularly influential role in the lives of same-sex families. As such confidentiality and deidentification of all results will be carefully considered in this project. Although contact information is necessary for linking parent and child responses to the survey and to follow-up families for further phases of research, contact details will be held in a separate password protected electronic database and can only be linked to responses, also password protected, by the use of a unique identification code. These passwords will only be known to two researchers.

As we will be asking about sensitive issues, including stigma and discrimination, we need to be aware of the possibility for psychological distress in our population. We will be providing contact details for gay and lesbian counseling services to help mitigate against this. Finally care must be taken in presenting our results. While we do not yet know what our research might show it is important to ensure that the results cannot be used to harm same-sex families in any way.

## Discussion

There are a number of limitations to ACHESS. The convenience sampling used for recruitment does allow for some bias in our sample; however this is not possible to overcome due to the hidden nature of the same-sex attracted population in Australia. To combat this, attention will be paid to the demographic characteristics of recruited participants with efforts made to target any under-represented groups that may emerge. Although we aim to achieve a large sample size overall the number of possible combinations of family types might make sub group analysis statistically underpowered. Grouping of responses may be necessary to allow for suitable analysis, however this will weaken the potential for inferences across the population as a whole.

ACHESS is the first quantitative Australian study to consider the complete health and wellbeing of children with same-sex attracted parents and its significance is in its contribution to the international literature on children from same-sex families. In particular it is, to our knowledge, the first study to take a holistic approach to child health and wellbeing and is aiming to be the largest study of its kind. We hope that this study will be able to fill many gaps in previous research and will provide the first insights into the health and wellbeing of children raised from birth by gay male parents.

By inviting participants to provide contact details to allow follow-up work we hope to be able to build this research further after the initial wave of surveys. It is anticipated that future work will involve qualitative sub-studies that will add a level of contextualisation that has often been missing from such research and by involving whole families it will provide a voice to children that is otherwise difficult to achieve.

Although the current study protocol is time limited, and as such requires a particular focus, it will be possible to launch further waves of research about children in same-sex families. Building on the rich detail that the ACHESS project provides, as described in this study protocol, it will then be possible to explore questions about child health and wellbeing in more depth.

## Abbreviations

ACHESS, The Australian Study of Child Health in Same-Sex Families; BSS, Bos Stigmatisation Scales; CBCL, Child Behaviour Checklist; CHQ, Child Health Questionnaire; K10, Kessler Psychological Distress Scale; ITQOL, Infant Toddler Quality of Life Survey; SDQ, Strengths and Difficulties Questionnaire.

## Competing interests

There are no financial competing interests. SC, RM and JP have links with community support groups for same-sex parent families.

## Authors' contributions

SC conceived of the study, co-ordinated the research, identified the relevant background material, designed the survey instrument and overall project structure, and drafted the manuscript. EW assisted with the study design, in particular elements relating to measures of child health and wellbeing, and contributed to the development of the manuscript. RM assisted with study design and refinement, contributed to the background work and provided extensive insights into the manuscript. JP made substantial contribution to study design, in particular participant recruitment and relevant items in the survey instrument, and contributed to the development of the manuscript. ED provided assistance with the study design, technical input on survey development, and contributed to draft of the manuscript. All authors read and approved the final manuscript.

## Pre-publication history

The pre-publication history for this paper can be accessed here:

http://www.biomedcentral.com/1471-2458/12/646/prepub
